# The Energy & Raw Materials Factory: Role and Potential Contribution to the Circular Economy of the Netherlands

**DOI:** 10.1007/s00267-018-0995-8

**Published:** 2018-01-30

**Authors:** Kees van Leeuwen, Eli de Vries, Stef Koop, Kees Roest

**Affiliations:** 1 Copernicus Institute for Sustainable Development and Innovation, Utrecht University, Heidelberglaan 2, Utrecht, 3584 CS The Netherlands; 2 KWR Watercycle Research Institute, Groningenhaven 7, Nieuwegein, 3433 PE The Netherlands; 3 Ministry of Infrastructure and Water Management, Rijnstraat 8, The Hague, 2515 XP The Netherlands

**Keywords:** Circular economy, Resource recovery, Wastewater treatment, Energy recovery, Bio-ALE, Bioplastics

## Abstract

Water is an abundant resource worldwide, but fresh and clean water is scarce in many areas of the world. Increases in water consumption and climate change will affect global water security even further in the near future. With increasing numbers of people living in metropolitan areas, water, energy, and materials need to be used carefully, reused and renewed. Resource scarcity is the driver behind the circular economy. The recovery of materials and energy can add significant new value streams and improve cost recovery and water quality. In this paper, we present the creation of the Energy & Raw Materials Factory (ERMF) of the Dutch Water Authorities, also known as the Resource Factory, as one of the solutions to this global challenge of water in the circular economy. Resources like cellulose, bioplastics, phosphate, alginate-like exopolymers from aerobic granular sludge (bio-ALE), and biomass can be recovered. Bio-ALE is an alginate-like polymer of sugars and proteins and can be used in agriculture and horticulture, the paper industry, medical, and construction industries. The ERMF demands significant investments but the return on investment is high both from a financial and environmental perspective, provided that markets can be realized. Experiences in the Netherlands show that the concept of the ERMF is viable and adds to the creation of a circular economy. Achieving climate neutrality and production of new and promising resources like bio-ALE are possible. The ERMF can contribute to the sustainable development goals (SDGs) of the United Nations on water and sanitation, once fully operational.

## Introduction

### Water in the Circular Economy

Water is an abundant resource worldwide, but fresh and clean water is scarce in many areas like in Sub Sahara Africa, India, parts of China, and the Middle East (Koop and Van Leeuwen 2017). Urbanization, the rapid increase of water consumption, as well as climate change will increase the importance of water security even further in the very near future (Villarroel Walker et al. 2017; Koop and Van Leeuwen 2017; Feingold et al. 2017). According to UNESCO, 2.1 billion people gained access to improved sanitation facilities since 1990, 2.4 billion still do not have access to improved sanitation and nearly 1 billion people worldwide still practice open defecation (WWAP 2015). In another UNESCO report an overview is given of global water supply and water treatment (WWAP 2017). The quality of water supply and wastewater treatment differs significantly between countries (OECD 2015a, b, 2016; Gawlik et al. 2017). This implies that there is a large potential to reduce water consumption, improve wastewater treatment and recover wastewater resources such as nutrients, energy, and the water itself.

The importance of the Circular Economy is caused by the continuous increase of the world population. Presently over 7 billion people inhabit our globe and in a few decades from now, this number will be 10 billion (UN 2015). More so, living standards and consumption are increasing at the same time. Due to these important developments, reserves of resources are getting smaller (EMF 2015; Mo et al. 2009; McDonough and Braungart 2002). An example is phosphate that is on the EU list of critical raw materials (European Commission 2014).

The reuse of wastewater should not be limited to water reuse only. Wastewater contains a lot of valuable resources (Holmgren et al. 2016; Villarroel Walker et al. 2014; Truffer et al. 2013). With increasing numbers of people living in metropolitan areas, water, energy, and materials need to be used carefully, reused and renewed. Resource scarcity necessitates the recovery of nutrients (like phosphorus and nitrogen), energy and other resources and can reduce maintenance cost, and add new value streams to improve overall cost recovery (WWAP 2017; Holmgren et al. 2016; Koop and Van Leeuwen 2017; Morée et al. 2013). This puts water where it belongs: in the broader context of the circular economy. In fact, drinking water and improved sanitation, health and food security are related and among the most important sustainable development goals (SDGs) of the United Nations (UN 2017).

Circular economy is a European goal (European Commission 2015). The mission of the Dutch government has been laid down in the National Circular Economy Program (Ministry of Infrastructure and the Environment 2016). Its goal is to realize sustainable economic growth by combining ecology and economy following a transition from a linear to a circular economy (PBL 2016). During the last decade, impressive technological progress has been made to transform waste into valuable resources. A circular economy can be realized in close cooperation between government, science and commercial companies.

The transition to a circular economy will only be possible when further innovations are made in many different technical processes (Frenken 2017a, b; Hekkert et al. 2007; 2011; Truffer et al. 2013), socio-technical transformation processes (Smith and Stirling 2010) together with improvements in governance (OECD 2015a, b; Koop et al. 2017; Kisparsky et al. 2013). Reduction of waste can take place in different ways as described in an update of Lansink’s waste hierarchy (Lansink 1979) with the best possible option at the top, i.e., by reduction followed by reuse, recycling, and energy generation. Incineration and landfills are less attractive from a circular economy point of view. In the Netherlands, a vision for wastewater for 2030 has been presented (Römgens and Kruizinga 2014; STOWA 2013).

### Technological Innovation and Green Deals

The Dutch Government has developed a policy of Circular Economy in 2016. This policy implies a structural approach to make production processes as circular as possible. In this policy, five production sectors are discerned as focus points, i.e. construction, plastics, consumer goods, industry, and organic waste. Also, specific subjects are discerned with regard to these sectors, e.g., cooperation, innovation, legislation, finance, and PR. Organic waste encompasses different types of waste, e.g., manure, green waste, and municipal and industrial wastewater. Due to the invention and application of sewers about 150 years ago (Geels 2005; 2006), a lot of organic waste is present in these waste streams. As a result of the collection and treatment of wastewater different valuable resources can be produced and recovered.

In the future transitions four elements are important: interaction, institutions, technology, and functions. According to Hekkert et al. (2007), interaction addresses the involved actors, their interests and their cooperation. Institutions regard especially legislation, regulations, and financial constructions. Technology concerns the state of development of a certain technology. Functions are less trivial. This element encompasses seven components, i.e., experimenting organizations, knowledge creation, knowledge diffusion, market development, guidance, financial means, and resistance. All these aspects are relevant to the Energy & Raw Materials Factory (ERMF) as discussed by Hekkert et al. (2007; 2011).

At different Universities in the Netherlands, water management and water treatment are important subjects due to the dependency of water in the Netherlands. The Technical University of Delft, Wageningen University & Research, Wetsus, Utrecht University Copernicus Institute for Sustainability, KWR Watercycle Research Institute, the domain Applied and Technical Sciences of the Netherlands Organization for Scientific Research, the Foundation for Applied Research for the Water Authorities (STOWA), and different commercial companies collaborate in scientific research dedicated to develop, test and apply new technologies for water treatment and resource recovery. Many prices and patents have been acquired to register the processes, techniques, and devices. The water authorities cooperate intensely in order to combine knowledge, interests, and efforts. As soon as a promising technology has been developed other participants are allowed to apply it. Dissemination of insight and knowledge in this part of the society has worked very well. This can be considered as a typical example of scaling up important innovations.

In order to coordinate the different research and development activities, the Foundation for Applied Research for the Water Authorities developed a special R&D plan for a period of 3 years (STOWA 2015). The total budget including subsidies comprises €60 million and most of it is financed by the water authorities in the ERMF and involved companies and knowledge institutes.

In order to stimulate innovations, different Green Deals have been signed respectively in 2012 for energy and phosphate, in 2014 for cellulose, bioplastics, phosphate, bio-ALE (Alginate-Like Exopolymers), biogas and CO_2_, and in 2016 for energy like solar energy, wind energy, and geothermal energy. A Green Deal is an agreement in which government, scientific institutions, and commercial companies create and stimulate innovations. This so-called triple helix of knowledge institutes and business-government relations is an internationally recognized model for innovation and socio-economic development. At this moment, over 200 Green Deals have been signed in the Netherlands (Green Deals 2017).

The results of these Green Deals will be beneficial to the water authorities but can also be used for e.g., the agro-food sector. The different water authorities each have their specific history, area, opportunities, and ambitions. This leads to different approaches with regard to resource recovery. Some water authorities can be considered as early adaptors, others are more in the rear of the peloton and the implementation process follows an S-shaped adoption curve like most other innovations.

One of the key principles of public administration policy is to offer collective goods and services for the lowest price possible and to implement the SDGs (UN 2017). This means that processes need to be evaluated on cost-effectiveness and social responsibility. Resource recovery from sewage sludge is expected to be an option to achieve this objective, but currently the financial resources needed to accomplish this are high. Further technological innovations are necessary to realize this goal. Due to the activities of the ERMF, a virtual organization of all water authorities, the recovery of energy and resources is increasing.

The focus of this article is (1) to predict the volumes and values of the produced resources from wastewater in the next decade and (2) to reflect on the innovation and governance aspects needed for this transformation. Data of Waternet, the water utility of Amsterdam and its surroundings, has been used to describe the process and to provide predictions at the national scale (e.g., Van der Hoek et al. 2016; 2017).

## The Energy and Raw Materials Factory

### The Process

Water authorities in the Netherlands no longer regard wastewater as merely a waste to be treated and processed, but as a valuable source of renewable energy, raw materials, and clean water. In order to contribute to the transition to a circular economy, the water authorities have set up a collaborative network organization called the ERMF.

The Netherlands presently encompasses 21 water authorities. These governmental institutions are autonomous and are legally responsible for management and quality of water in their area including treatment of municipal wastewater. In order to comply with the national policy plans towards a circular economy, they focus on the recovery of at least five resources from municipal wastewater: cellulose, bioplastics, phosphate, bio-ALE, and biomass. This development has started some years ago.

The ERMF is a complex, time demanding, and an expensive operation, as different options of energy and raw material production are explored. Struvite, biogas and cellulose are already produced. Until now ERMF achieved great success (ERMF 2017), but further development will be a long-term implementation challenge. During such a period, predicted volumes and prices can easily change due to all kinds of developments such as new entrants, price policy, and new products. Volumes and prices are indicated on the basis of the present day knowledge and expectations. This means that the business cases may change both in a positive or negative fashion. An interesting aspect is that the Water Authorities are governmental institutes. This makes it more difficult to sell products on the market. On the other hand this will create commercial options for other parties, separately or linked to the Water Authorities.

At this moment about 100 biogas installations, 12 phosphate installations, and 2 cellulose recovery installations are operational in the Netherlands (ERMF 2017). Currently, the water authorities use the generated energy by biogas to supply 30% of their need for electricity and this capacity will increase in the near future. The ERMF is a learning-alliance of the participants. It follows a learning-by-doing approach. Gaining practical experience is important as it will allow for future fine-tuning, adaptation, and anticipation regarding techniques, volumes, applications, markets, and legislative barriers. Many of the Sewage Treatment Plants (STPs), especially the bigger ones, will transform from traditional utilities into resource factories and can contribute significantly to achieving circular economies. The experiences of the ERMF may provide proactive utilities and engineering industries across the globe with business opportunities regarding design, construction, exploitation, and maintenance.

### The ERMF: Products and Markets

With regard to recovery of resources from sewage, there are different options, paths, and priorities. The current focus is on cellulose, bioplastics, phosphate, and bio-ALE. Cellulose can be recycled from toilet paper. This recovered cellulose can be used in road construction but further markets still have to be developed (Arcadis 2017). Bioplastics are formed by the use of a complicated process during which volatile fatty acids (VFAs) are firstly produced and these VFAs are later fed to microbes which form the wanted building blocks for bioplastics. This can be considered as a form of up-cycling. Phosphate can be recovered as e.g., struvite (MgNH_4_PO_4_·6H_2_O). In this way, phosphorus becomes available again since the recovered struvite can be applied as fertilizer. The extraction of bio-ALE from aerobic granular sludge can be considered as up-cycling due to the fact that a more valuable product is produced from waste. At the end, the organic waste stream can be digested into biogas (energy). Energy is at the base of the hierarchy of Lansink (1979). The value of a resource in the circular economy is determined by the degree to which a recovered resource means an up-cycling of the original resource.

The creation of an ERMF is capital intensive; the total number of STPs in the Netherlands is about 350. Of this number, some are quite big especially in the big cities and others in more rural areas are much smaller. Economies of scale can play an important role with regard to the cost of recovered resources. This implies that ERMFs will probably be created in big cities first. When experience has been gained and the costs of installations have dropped, it is expected that Resource Factories will also be built in smaller cities. The different resources are in different stages of development. Normally the stages of pre-development, development, take off, acceleration, and stabilization are discerned. In the ERMF some resources are in take-off stage such as cellulose, bio-ALE, and e.g., phosphate is in an acceleration stage. Biogas from digestion is in the stabilization stage, whereas bioplastics are in the early stages of development.

The present and expected number of installations, quantities, and values of the different recovered resources, described above, is presented in Table 1. The total value of the recovered resources for the Netherlands is potentially about €230 million per year or accumulated €2.3 billion in 10 years from 2030 (Table 1). Probably many STPs will have been transformed to ERMFs and markets for the recovered resources will be more developed by 2030.

**Table 1:** Number of installations, quantities, and values of recovered resources in 2018 and 2030 for the Netherlands. Data have been gathered based on interviews with Dutch water experts

Resource	Installations	Quantities 2018	M€ 2018	Quantities 2030	M€ 2030
Biogas	100	120 million m^3^	24^a^	200 million m^3^	40^a^
Phosphate	12	4 Kton	2^b^	20 Kton	8^b^
Cellulose	2	5 Kton	0	50 Kton	15^c^
Bio-ALE	10	0 Kton	0	85 Kton	170^c^
Bioplastic	1	–	–	–	–
Total			26		233

^a^Preliminary estimate. Biogas will mainly be used by the producers and utilities for own energy use

^b^Estimate based on total revenues including reduction of maintenance costs

^c^Currently there are 10 Nereda plants that can potentially produce Bio-Ale. Estimates for cellulose and Bio-Ale are based on input from Waternet and Royal HaskoningDHV experts

#### Biomass and biogas

Two promising sales routes have been developed for biomass from water systems: biomass as soil improver and biomass as fiber for the paper and cardboard industry. The sales route as soil improver reduces the costs that the water authorities now have on the processing of clippings, halving these costs. The second route, biomass as a fiber for the paper and cardboard industry, can create a higher value for the biomass. This means that transport of biomass can take place over longer distances and water boards (and other green managers) can achieve efficiency through cooperation (Arcadis 2017). Currently, no economic values can be added to these options. In this section, however, the focus will be on the production of biogas.

In the Netherlands, approximately 100 digesters are operational and biogas (a mixture of CH_4_ and CO_2_) is produced. The current production of biogas is 116 million m^3^ per year. With a price of € 0.20 per m^3^, this is a revenue of €24 million per year. This covers almost 40% of the energy consumption of the water authorities (Van Nieuwenhuijzen et al. 2016). This is in line with the 107 million m^3^ per year as reported by Statistics Netherlands for 2015 (Statistics Netherlands 2017). Interviewed experts have estimated future biogas production in 2030 at 200 million m^3^ per year leading to total revenue of €40 million per year. The maximum potential for biogas production from STPs is higher. For 2020 and 2030 this has been estimated at 195 and 438 million m^3^, respectively (Routemap Green Gas 2014). In the near future, the need for energy for wastewater treatment might drop significantly due to the introduction of e.g., the Nereda process. In fact, it can be expected that the water authorities will be self-supporting regarding energy, when they combine it further with wind, solar, and geothermal energy, or even become net producers of energy as in the case of the city of Hamburg (Van Leeuwen and Bertram 2013).

From Table 1 it can be seen that the production of biogas is of main importance. Though the biogas production will increase in the coming years, the production of other resources will develop significantly. This goes especially for bio-ALE which is at this moment in the infancy phase and might represent more than 50% of the total value of the production of the ERMF in the year 2030. An important aspect of bio-ALE is that it is too complicated to produce it chemically which enhances the market position.

#### Phosphate

Phosphate causes eutrophication of surface waters (Morée et al. 2013; Koop and Van Leeuwen 2017) and, at the same time, is a scarce resource appearing on the European Critical Raw Materials list (European Commission 2014). On the global scale phosphate reserves are limited and especially found in Morocco and China. Phosphate is mainly used for the production of fertilizer for agricultural purposes. Currently, 12 phosphate recovery installations are operational at municipal STPs. Influents of STPs contain approximately one kilo P per person per year. This is approximately equal to 20,000 tons per year in the Netherlands.

Recently, Waternet introduced a new nutrient recycling facility to treat wastewater for the City of Amsterdam and other communities, which can produce 900 tons of struvite (MgNH_4_PO_4_·6H_2_O), which contains 28% PO_4,_ on an annual basis (Van der Hoek et al. 2017). Struvite is formed in digested sludge based on a process with the addition of magnesium chloride (MgCl_2_) and aeration with compressed air to raise pH. The plant treats approximately 2000 m^3^ sludge per day. The investment costs are €4 million, while the expected savings are €400,000 per year (Van der Hoek et al. 2017). The savings of this process consists of selling of the struvite, lowering the maintenance costs and better sludge dewatering. Extrapolated for the Netherlands the generated return would be approximately €8 million per year.

#### Cellulose

Cellulose is a material which ends up in sewage water due to the use of toilet paper. A person uses on average of 12 kg of tissue paper per year (Ruiken et al. 2013), which means that the total potential volume is approximately 200,000 tons per year in the Netherlands. Nevertheless, not all tissue paper can be recovered at STPs. An estimate of 25% is assumed which will lead to a maximum of 50,000 tons per year for 2030. The current recovery with two installations is about 5000 tons per year. Fine-sieving of cellulose is expected to take place when more capacity of STPs is required and may increase the capacity of STPs by about 10% (Roest 2017). Although there are currently no revenues from cellulose recovery at all, as considerable investments are necessary, the potential revenue is interesting due to expected price of this material, i.e., approximately €300 per ton. In conclusion, the current total value of the recovered cellulose is negligible, but further developments may create revenues of €15 million per year by 2030.

#### Alginate-like exopolymers (bio-ALE)

Alginate is a naturally occurring anionic polymer. It is a polysaccharide, typically obtained from brown seaweed and has been extensively investigated and used for many biomedical applications which include wound healing, drug delivery, and tissue engineering due to its biocompatibility, low toxicity, and relatively low cost. Alginate also serves as a gelling agent in textile printing, food preparation, and paper industry (McHugh 1987; Lee and Mooney 2012).

**Fig. 1:**
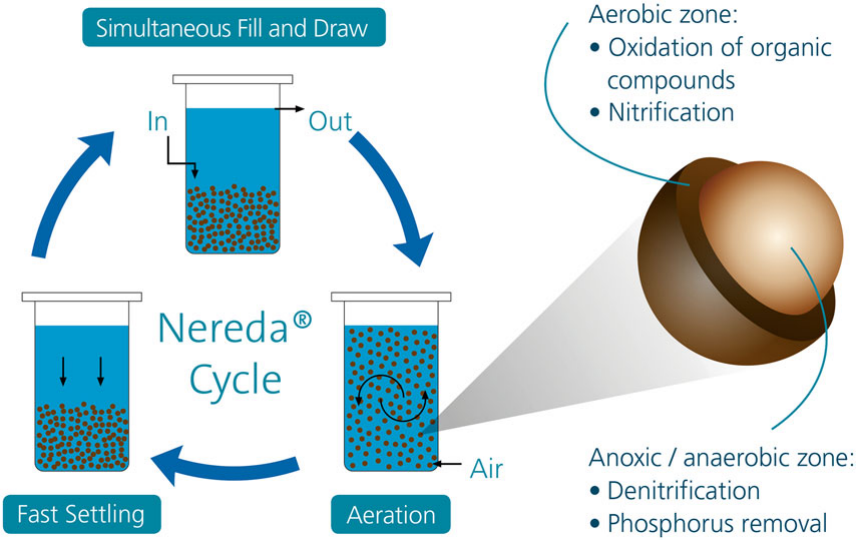
The Nereda process is an aerobic granular sludge technology that purifies wastewater by controlling the growth and formation of microorganisms. The Nereda process reduces energy up to 50% and the needs for space by 70%, involves no chemicals and yields a component with similar characteristics as alginate, i.e., bio-ALE (*Source*: Royal HaskoningDHV, with permission)

The Nereda aerobic granular sludge technology (Fig. 1) produces bio-ALE from aerobic granular sludge (Van Der Roest et al. 2015; Hogendoorn 2013; Royal HaskoningDHV 2017a). The Nereda process was developed at the Delft University of Technology through a public-private partnership with the Dutch Foundation for Applied Water Research (STOWA), six Dutch water authorities and Royal HaskoningDHV, the company exploiting the technology globally. The process combines several steps of the traditional wastewater treatment process in one reactor and reduces energy and space requirements significantly (Royal HaskoningDHV 2017b).

Bio-ALE is an alginate-like polymer of sugars and proteins and can be used in agriculture and horticulture, the paper industry, medical, and construction industries. Bio-ALE can be applied as fibers, gel or foam. Fibers can be used for the production of absorbing tissues, gels can be used as glue for production of fertilizer pellets and foam can be used for the production of fire resistant boards. The liquid version can be used for thickening inks, improving paper quality, increasing quality of curing concrete. (Hogendoorn 2013; Royal HaskoningDHV 2017a). The first bio-ALE production installations will be built in Zutphen and Epe in 2019 in the Netherlands. In a field test it was demonstrated that 18 kg bio-Ale can be produced from 80 kg of Nereda granular sludge (Royal HaskoningDHV (2017a). It is expected that an industrial Nereda alginate factory like the one in Zutphen will produce about 400 tons of alginate per year (Royal HaskoningDHV 2017a). The total Dutch production is estimated at 85,000 tons per year from 2030. The price depends on the quality and the total value is currently estimated at €170 million per year from 2030. Nerada installations can readily adopt a bio-ALE production based on the test results in Zutphen. Currently 10 Nereda installations are operational in the Netherlands and about 40 installations worldwide.

#### Bioplastic

Bioplastic can be produced from sewage sludge. There have been some experiments and tests. The production cost of this material, however, are currently still rather high, it is twice as much as the regular market price. Furthermore, there is no available stable industrial production process yet. The PHARIO project in the Netherlands aims at the production of bioplastics (polyhydroxyalkanoates) from sewage.

The realization of the ERMF will lead to new relations with other organizations. If coordination and cooperation of activities are possible, significant economies of scale can be achieved. The ERMF will have relations with companies interested in resource recovery. Phosphate can be used as fertilizer. Bio-ALE is very versatile due to its many market possibilities. Biogas can be used for internal purposes by the use of combined heat and power (CHP) generation in a gas engine. In this way, electricity and heat are produced as well as CO_2_. The sales of produced resources can be done collectively as is done by the Dutch drinking water companies (AquaMinerals).

### Water Management and Governance of Amsterdam

In order to describe the effects of the ERMF in the Netherlands, Amsterdam is taken as a case study. There are four reasons for this: (1) Amsterdam is the largest city in the Netherlands with currently over 853,000 inhabitants, (2) Amsterdam is served by one municipally owned utility for water called Waternet which manages the entire urban water cycle, including drinking water, water safety, surface water and wastewater transport, and treatment. This integrated approach and close ties with the municipality and regional water board provides the right innovative climate for the recovery of energy and raw materials, (3) Amsterdam performs very well on many challenges with regard to water, waste, and climate change. The city has the highest score in the Blue City Index (BCI) of all cities assessed so far. The BCI is the geometric mean of 25 indicators of the City Blueprint Framework (Koop and Van Leeuwen 2015; Gawlik et al. 2017; Feingold et al. 2017) and, (4) Amsterdam performs very well on water governance. This is explained in more detail below.

**Fig. 2:**
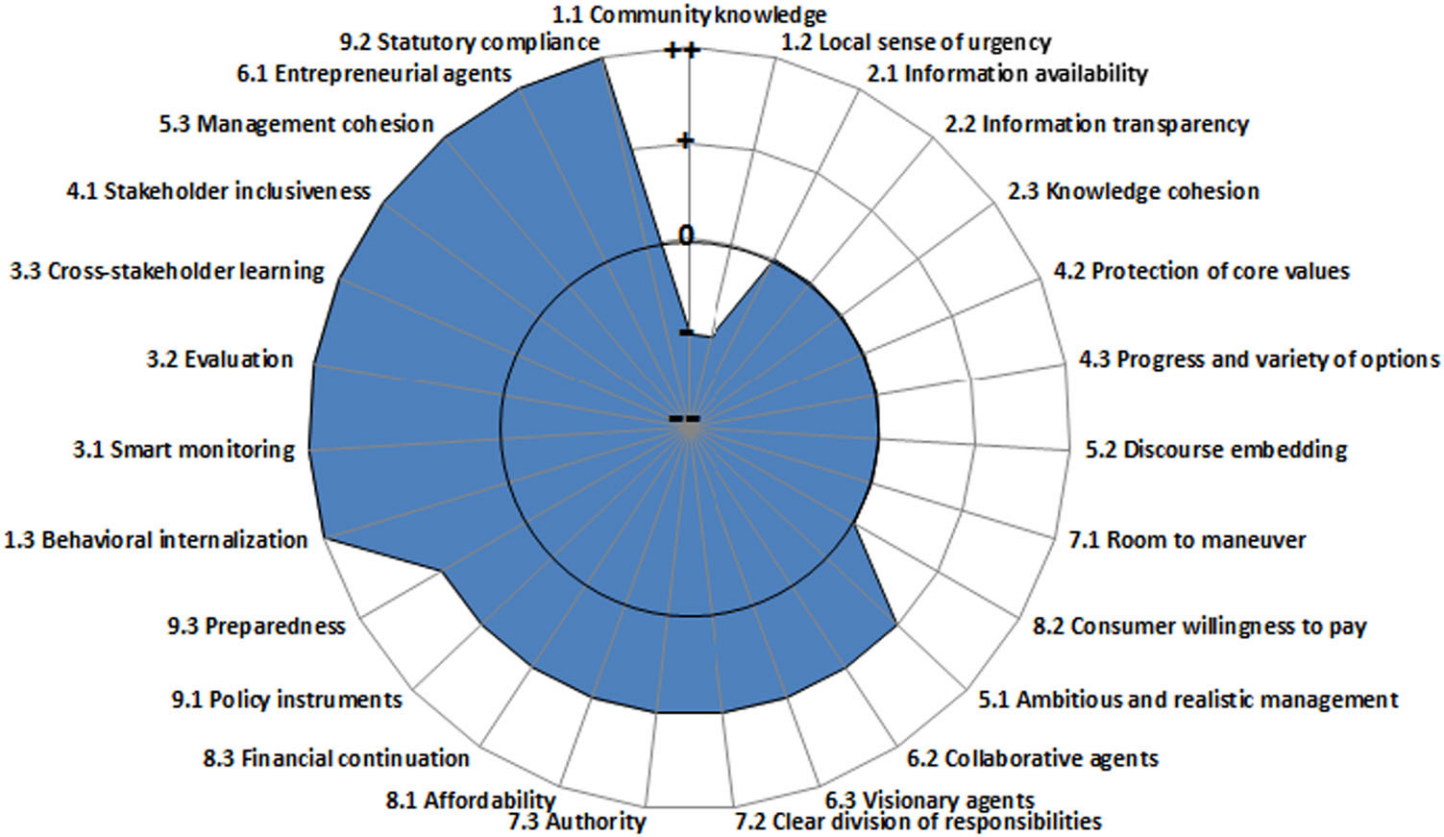
Scores of the 27 indicators of the GCF water governance performance on wastewater treatment for the city of Amsterdam (Koop et al. 2017)

Water management is a technological challenge, but also a governance challenge (Hendriks 2014; OECD 2015b; 2016; Koop and Van Leeuwen 2017). This is the reason why a comprehensive governance capacity assessment framework for cities has been developed. Koop et al. (2017) proposed an empirical-based diagnostic governance capacity framework (GCF) that provides insights into the main conditions that together determine the governance capacity of all different stakeholders involved in the governance of a water-related challenge, e.g., wastewater treatment. This GCF consists of 27 governance indicators that are scored according to a Likert scale ranging from very encouraging (++) to very limiting (−−). A detailed description of the scoring system is available (EIP Water 2017). The challenge of achieving a circular economy transcend administrative boundaries and challenges the government, local authorities, the private sector, and all other stakeholders to explore new ways of cooperation, adopt new strategies, evaluate and learn from them, adjust policy and improve implementation repeatedly. In other words, it requires sufficient governance capacity to innovate and implement ERMFs. Amsterdam’s capacity to govern its wastewater treatment is well-developed (Fig. 2; Koop et al. 2017).

The circular economy approach has proven to be beneficial in Amsterdam, especially Waternet’s initiative on integrated water cycle management. The results indicate that Amsterdam has a few favorable governance conditions that encourage continued innovation and the implementation of resource recovery from wastewater. Existing practices include material recovery from wastewater, e.g., struvite, energy recovery from the water cycle, energy production from wastewater, such as biogas production, incineration of sludge, and solid waste (Van Leeuwen and Sjerps 2015; Van der Hoek et al. 2017). Many of these projects are carried out in cooperation with different actors. This transition enabled cost savings and operational efficiency (Van der Hoek et al. 2016).

The availability of sufficient financial resources (indicator 8.3) has successfully ensured a continued R&D amongst various research institutes in cooperation with practitioners. The level of monitoring (indicator 3.1), evaluation (indicator 3.2), and cross-stakeholder learning (indicator 3.3) has contributed to management improvements and research innovations. In particular, the cooperation between research institutes and practitioners has enabled continuous innovation that can be applied in STPs. Besides the technical aspects of resource recovery, Amsterdam also actively explored ways to create new markets for these resources such as for struvite (Van Leeuwen and Sjerps 2015; Van der Hoek et al. 2016, 2017). Governance indicators that may limit the overall governance capacity for Amsterdam on wastewater treatment are (1.1) community knowledge and (1.2) local sense of urgency. The understanding of causes, impact, scale, and urgency of wastewater treatment and resource recovery is relatively low amongst citizens and local communities.

## Discussion

Water, waste, and climate change are important challenges that may affect global social stability. Most of our global challenges, i.e., the Sustainable Development Goals can best first be addressed at the level, where these problems will concentrate: in cities (Katz and Bradley 2013; Koop and Van Leeuwen 2017). These macro-developments will be important in the transition and system change as we currently observe for water and waste in the context of the circular economy, as has also been observed by Geels (2005, 2006) for water supply and hygiene in the Netherlands in the period 1850–1930.

Resource recovery is far more than just a matter of technology. The ERMF as presented in this paper is an attempt to implement water in the circular economy. The ERMF offers the possibility to make use of valuable resources present in sewage. This creates a new level in society, i.e., the recovery of essential resources from waste (Braungart et al. 2007). This holds especially for bio-ALE. This resource is versatile and can be used for improving the quality of paper and thickening inks, pasta, and gels. It can also be used for improving the curing of concrete and the production of fertilizer and be applied in fire resistant boards and materials for construction and aerospace industry. Production in the form of fibers opens up the possibility to make fluid absorbing tissues. This implies that bio-ALE can play an interesting and useful role in other sectors of the Circular Economy, i.e., industry, consumer goods, plastics, and construction. The availability on a broad scale for a low price of bio-ALE can mean an important step ahead in industrialized society as well as in developing societies. The production of bio-ALE, however, demands large investments in facilities and a well-developed governance system regarding water supply and water treatment.

The volumes and prices mentioned in this article are provided by a few interviewed Dutch experts and a few sources of literature. During the completion of the ERMF, changes may occur that can change volumes and market conditions. This may result in significant changes in the business cases. Bio-ALE is a potential important product and could comprise 50% of the turnover of the ERMF. Due to the many applications of bio-ALE and the importance of its applications it may be expected that price erosion will not occur. Biogas represents an important value but is used for internal purposes so real price erosion is not very foreseeable.

All these developments demand a significant investment of the different Water Authorities. A payback period of 10 years is commonly used regarding sustainability investments. The estimated prices can only be obtained when a constant generation of high-quality products can be guaranteed. Otherwise, customers will soon lose their interest, prices will then drop, and markets will be lost. The operating expenditures and capital expenditures are difficult to estimate at this stage. The annual revenues of the ERMF, calculated on the basis of the number of inhabitants in the Netherlands (17,000,000) can be estimated at approximately €14 per person per year in 2030, but this will require at least similar-sized investments upfront in the ERMF. The relocation of old STPs or the construction of e.g., new Nereda installations that require less (expensive) space, may lead to great financial benefits, although they are difficult to quantify at this moment. Assuming that all the necessary investments for ERMFs are provided and the revenues of €14 per person per year are extrapolated to a global population of 7 billion people, the total revenues will be about €100 billion per year. The ambition articulated in the SDGs on water and sanitation (UN 2017) has recently been estimated at US $114 billion per year up to 2030 (World Bank 2017).

In the Netherlands, Water Authorities are governmental and autonomous institutions. This implies that they are not driven by profit maximization. There are differences in the way the 22 different Water Authorities implement this concept and at what speed, but the combination of efforts and the stimulation of cooperation is helpful in accelerating the implementation of the ERMF. Furthermore, this might increase economies of scale, which can lead to lower cost for the individual participants.

The recovered resources are derived from waste and have still a ‘waste label’. National and European efforts are in place to remove the waste label from these resources to create a real circular economy. For resources which are created and used within the company, the consequences are manageable, but for recovered resources with external applications, this is a major issue. So for cellulose, phosphate, and bio-ALE, the potential contamination with pathogens, pharmaceuticals, hormones, pesticides, antibiotics, and industrial chemicals, including their degradation products, plays a role and are major societal and political concerns.

Combining good governance with new technologies in a timely fashion may create new opportunities and improve cost-effectiveness needed as water treatment and water infrastructure is expensive (UNEP 2013). The governance capacity analysis of Amsterdam showed a low-level awareness. This may limit further support for the allocation of financial resources, actions and policies needed to optimally exploit the available resources in wastewater. Hence, the major challenge is to communicate the relevance and necessity of circular solutions to reduce our waste production, decrease resource dependency and lower greenhouse gas emissions. Therefore, resource recovery from wastewater is also a major communication challenge. This applies to other fields and sectors as well. For the implementation of a circular economy we need to explain how this concept—through many circular solutions in many different sectors—can benefit major societal issues such as water and food security (Koop and Van Leeuwen 2017). Furthermore, water in the circular economy provides profitable investment opportunities as well.

## Conclusions

1. The ERMF enables the recovery of clean water, cellulose, bioplastics, phosphate, bio-ALE, and biogas from municipal wastewater. The value of the recovered resources including the reduction of maintenance cost in the Netherlands is estimated at €233 million per year from 2030. This is approximately €14 per person per year. Similar investments are needed to create ERMFs.
2. Assuming that all the necessary investments for ERMFs are provided and the revenues of €14 per person per year are extrapolated to a global population of 7 billion people, the total revenues will be about €100 billion per year. The ambitions articulated in the SDGs on water and sanitation (UN 2017) have recently been estimated at US $114 billion per year up to 2030 (World Bank 2017). The circular economy (introduction of ERMF at global scale) and the UN SDGs on water and sanitation provide potential win-win’s.
3. Collective activities have to be developed to create markets and to sell the recovered resources in order to prevent unwanted competition between water authorities. The realization of the ERMF demands a long-term strategy, i.e., a long lead time, significant financial resources, and continuous attention from all stakeholders.
4. Cross-institutional collaboration and communication is fundamental. We found that stable collaborative networks, alignment between research and practice, and well-established monitoring and evaluation, are important conditions that increase the long-term capacity to establish and further develop the recovery of resources from wastewater.
5. There are commercial opportunities for companies that are active in the engineering of water treatment installations. They can play an important role in creating Resource Factories, both at the national, European, and global level.
